# Endothelial Nitric Oxide Synthase Prevents Heparanase Induction and the Development of Proteinuria

**DOI:** 10.1371/journal.pone.0160894

**Published:** 2016-08-09

**Authors:** Marjolein Garsen, Angelique L. Rops, Jinhua Li, Katrien van Beneden, Christiane van den Branden, Jo HM Berden, Ton J. Rabelink, Johan van der Vlag

**Affiliations:** 1 Department of Nephrology, Radboud university medical center, Nijmegen, The Netherlands; 2 Department of Anatomy and developmental Biology, Monash University, Clayton, Victoria, Australia; 3 Department of Human Anatomy, Liver Cell Biology Lab, Vrije Universiteit Brussel, Brussels, Belgium; 4 Department of Nephrology, Einthoven Laboratory for Vascular Medicine, Leiden University Medical Center, Leiden, The Netherlands; Temple University, UNITED STATES

## Abstract

Endothelial nitric oxide synthase (eNOS) deficiency exacerbates proteinuria and renal injury in several glomerular diseases, but the underlying mechanism is not fully understood. We recently showed that heparanase is essential for the development of experimental diabetic nephropathy and glomerulonephritis, and hypothesize that heparanase expression is regulated by eNOS. Here, we demonstrate that induction of adriamycin nephropathy (AN) in C57BL/6 eNOS-deficient mice leads to an increased glomerular heparanase expression accompanied with overt proteinuria, which was not observed in the AN-resistant wild type counterpart. *In vitro*, the eNOS inhibitor asymmetric dimethylarginine (ADMA) induced heparanase expression in cultured mouse glomerular endothelial cells. Moreover, ADMA enhanced transendothelial albumin passage in a heparanase-dependent manner. We conclude that eNOS prevents heparanase induction and the development of proteinuria.

## Introduction

Proteinuria is a key feature of many glomerular diseases and an independent risk factor for the progression to renal failure. Proteinuria is caused by damage to the glomerular filtration barrier, which is composed of glomerular endothelial cells covered by a glycocalyx, the glomerular basement membrane and podocytes. Several studies showed that a reduced glomerular endothelial glycocalyx thickness is associated with the development of proteinuria [1–3]. We recently showed that endothelial dysfunction and damage precedes podocyte damage in adriamycin-induced nephropathy (AN) [4], indicating that a healthy glomerular endothelium is important to prevent the development of proteinuria and renal damage.

Previous studies showed that a decreased nitric oxide (NO) production and availability contributes to endothelial dysfunction [5]. NO is produced by endothelial cells through endothelial NO synthase (eNOS). Multiple factors have been suggested to be involved in the regulation of eNOS, including reactive oxygen species (ROS), angiotensin II, asymmetric dimethylarginine (ADMA), protein kinase C (PKC), advanced glycation end products (AGEs), vitamin D and tumor necrosis factor (TNF)-α [6,7]. eNOS deficiency exacerbates renal injury in accelerated anti-glomerular basement membrane (GBM) glomerulonephritis [8], experimental focal segmental glomerulosclerosis (FSGS) [9], and diabetic nephropathy (DN) [10]. In addition, eNOS gene delivery prevented the development of proteinuria in a rat model of FSGS [11]. Recently, we showed that induction of AN in C57BL/6 mice, an AN resistant strain, with eNOS deficiency induced overt proteinuria, glomerulosclerosis, tubulointerstitial fibrosis and inflammation [4]. The mechanism how eNOS deficiency exacerbates proteinuria and renal damage remains unknown.

We previously showed that heparanase, a heparan sulfate (HS) specific endoglycosidase, is essential for the development of proteinuria and renal damage in experimental DN and glomerulonephritis [12,13]. In both aforementioned models heparanase-deficient mice displayed a preserved glomerular HS expression compared to WT mice. Notably, loss of glomerular HS expression is associated with the development of proteinuria in most human and experimental glomerular disease [14]. Heparanase is positively regulated by ROS, angiotensin II, aldosterone, AGEs, PKC, high glucose and TNF-α [13,15–18], and negatively regulated by vitamin D [19]. Interestingly, the aforementioned factors that regulate heparanase are also involved in regulation of eNOS and endothelial function. Importantly, glomerular endothelial glycocalyx thickness is reduced in diabetic eNOS-deficient mice compared with diabetic wild type (WT) mice [20]. In addition, heparanase has been shown to impair glycocalyx thickness [1,3]. Therefore, we hypothesize that heparanase expression is controlled by eNOS.

## Materials and Methods

### Animals

WT C57BL/6J mice (Monash Animal Services, Monash University, Australia) and eNOS-deficient mice in a C57BL/6J background (Jackson Laboratories, Ben Harbor, ME, USA) were housed and bred at Monash Animal Services. Animal handling and experiments were approved by the Monash University Animal Ethics Committee and according to the “Australian Code of Practice for the Care and Use of Animals for Scientific Purposes”. Balb/c mice (Harlan, Horst, The Netherlands) were housed at the “Vrije Universiteit Brussel” and animal experiments were approved by the Animal Care and Use Committee of the “Vrije Universiteit Brussel”.

### Adriamycin-induced nephropathy

AN was induced in 8-week-old C57BL/6J WT and eNOS-deficient mice by an intravenous injection of 10.5 mg/kg body weight of adriamycin (Sigma-Aldrich, St. Louis, MO, USA), as described previously [4]. Control mice received an equivalent volume of normal saline (NS). Mice were sacrificed 14 days after induction of AN. Five mice were used per group.

In Balb/c mice (8 weeks old), AN was induced by an intravenous injection with 10 mg/kg adriamycin (Pharmacia, Brussels, Belgium) as described [21]. Control mice were injected with NS. Mice, five-six per time point, were sacrificed 10 and 23 days after induction of AN.

### Immunofluorescence staining

Glomerular heparanase and HS expression was determined by indirect immunofluorescence staining on cryosections (2 μm) as described [22]. Primary antibodies included heparanase (HPA1, ProsPecTany, Rehovot, Israel) and a mouse monoclonal anti-HS antibody, JM403 [23]. Secondary antibodies included goat anti-rabbit IgG Alexa 488 and goat anti-mouse IgM Alexa 488 (Invitrogen Life Technologies, Breda, The Netherlands). Glomerular heparanase and HS staining intensities were scored in 50 glomeruli per section on a scale between 0 and 10 (0 = no staining, 5 = 50% staining, 10 = 100% staining). Scoring was performed by two independent investigators on blinded sections using a Leica CTR6000 microscope.

### Cell culture and transendothelial albumin passage

Conditionally immortalized mouse glomerular endothelial cells (mGEnC-1) were cultured as described previously [24]. Heparanase was stably silenced in mGEnC-1 by transfection of a heparanase shRNA construct (Qiagen, Venlo, The Netherlands) with Lipofectamine 2000 into undifferentiated mGEnC-1 and subsequent selection with G418 (Sigma-Aldrich). Differentiated mGEnC-1 were treated with the eNOS inhibitor ADMA (10 μg/ml; Millipore, Amsterdam, The Netherlands) for 18 hours. For transendothelial albumin passage, differentiated mGEnC-1 seeded on polyester membranes in tissue culture inserts (0.4 m pore size; Corning Incorporated, NY, USA) were treated with the eNOS inhibitor ADMA as outlined. Transendothelial albumin passage was determined as described previously [19].

### RNA isolation, cDNA generation and real-time PCR

Total RNA was extracted from mouse renal cortex and mGEnC-1 using the RNeasy mini kit (Qiagen, Venlo, The Netherlands). 1 μg RNA was reverse-transcribed into cDNA using the RevertAid First Strand cDNA Synthesis Kit (Thermo Scientific, Waltham, MA, USA). Heparanase mRNA expression levels were measured by real-time PCR using gene-specific primers (Isogen Life Science, de Meern, The Netherlands) and the Fast-start SYBR Green SuperMix (Roche Diagnostics, Mannheim, Germany), and analyzed with the CFX real-time PCR system (Bio-Rad Laboratories, Hercules, CA, USA). Used primers: Heparanase, forward 5’-GAGCGGAGCAAACTCCGAGTGTATC-3’, reverse 5’-GATCCAGAATTTGACCGTTCAGTT -3’, and glyceraldehyde-3-phosphate dehydrogenase (GAPDH), forward 5’-AGAAACCTGCCAAGTATGATGAC-3’, reverse 5’-TCATTGTCATACCAGGAAATGAG-3’. Heparanase gene expression levels were quantified with the delta-delta C_T_ method using GAPDH as the housekeeping gene.

### Statistical analysis

Values are expressed as mean ± SEM. Significance was evaluated by a one-way ANOVA and *post hoc* analysis with Tukey’s multiple comparison test. A Student’s *t*-test was used for comparison of expression between two different groups. A two-way repeated measures ANOVA with Bonferroni post-test was used to determine significant differences in transendothelial albumin passage. Statistical analysis was performed using GraphPad Prism 5.03 (GraphPad Software, Inc., San Diego, CA, USA). A P-value of ≤ 0.05 was considered statistically significant.

## Results

### eNOS prevents adriamycin-induced heparanase expression and proteinuria

**Fig 1:**
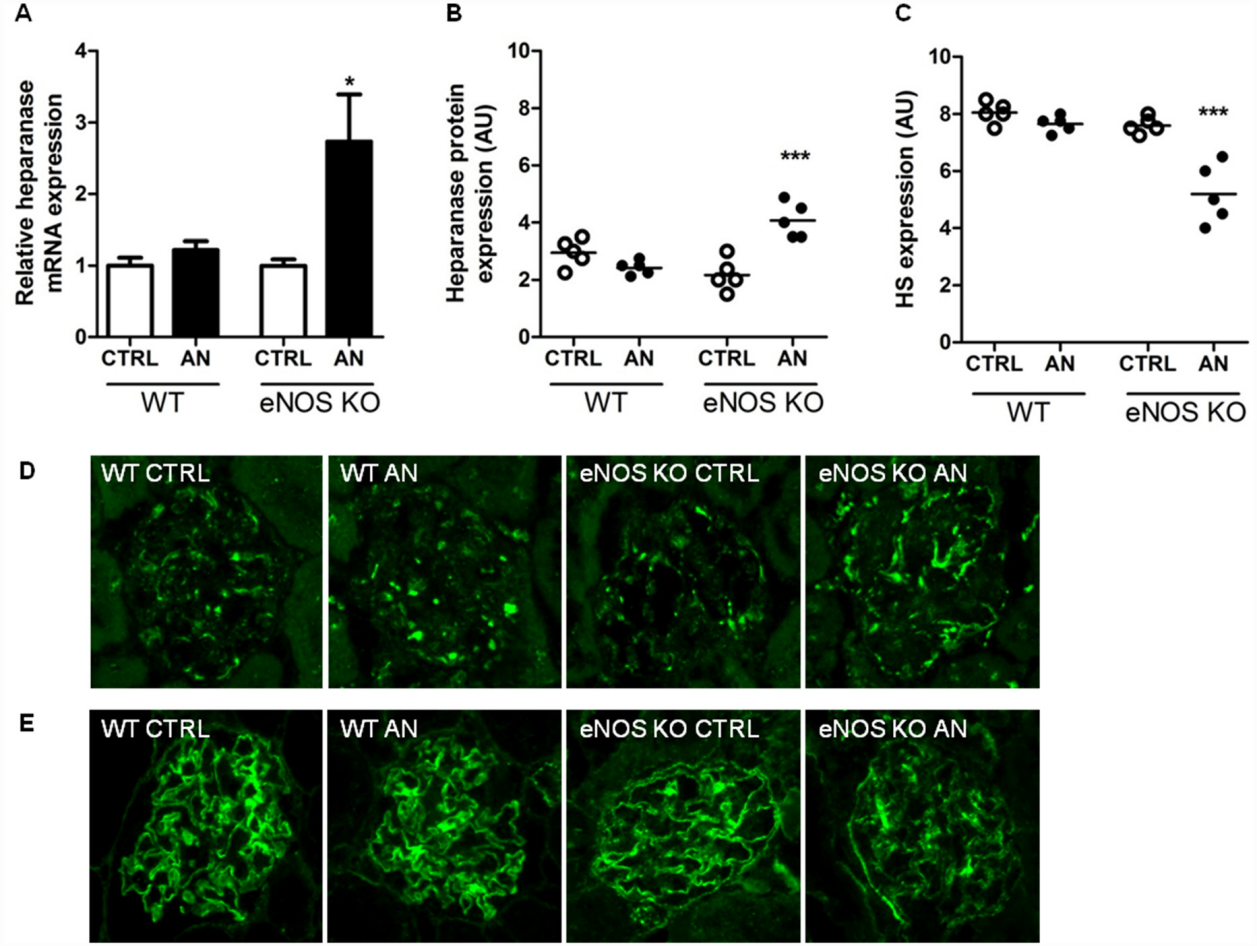
eNOS prevents adriamycin-induced heparanase expression and proteinuria. Adriamycin-nephropathy (AN) was induced in C57BL/6 WT mice, an AN resistant strain, and C57BL/6 eNOS-deficient mice. Mice were sacrificed 14 days after the induction of AN. By induction of AN in WT mice, (**A**) cortical heparanase mRNA expression, (**B**,**D**) glomerular heparanase protein expression, as determined by immunofluorescence staining, and (**C**,**E**) glomerular HS expression, as determined by immunofluorescence staining, were comparable to control. Cortical heparanase mRNA expression and glomerular heparanase protein expression were significantly increased in eNOS-deficient mice after induction of AN, whereas glomerular HS expression was reduced. (**D**) Representative pictures showing glomerular heparanase protein expression and (**E**) glomerular HS expression (magnification x400). 5 mice per group were used for analysis. *P<0.05 and ***P<0.001 versus CTRL. WT, wild type; eNOS KO, endothelial nitric oxide synthase-deficient; HS, heparan sulfate; CTRL, control; AU, arbitrary units.

**Fig 2:**
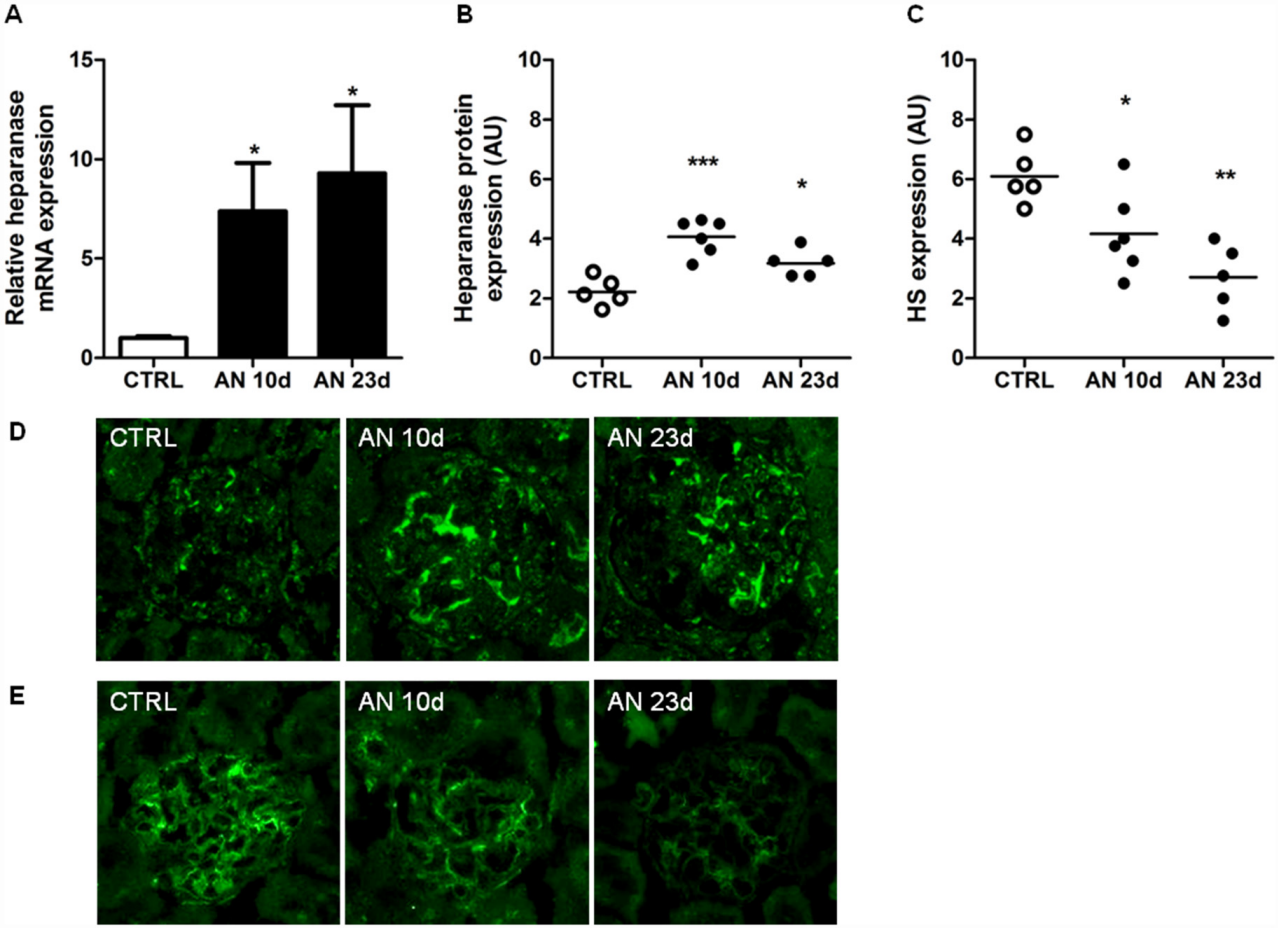
Heparanase expression was increased and HS expression reduced by induction of adriamycin nephropathy (AN) in AN-sensitive Balb/c mice. AN was induced in Balb/c, which are sensitive for AN-induced renal damage. Mice were sacrificed 10 and 23 days after the induction of AN. By induction of AN, (**A**) cortical heparanase mRNA expression and (**B**,**D**) glomerular heparanase protein expression, as determined by immunofluorescence staining, were significantly increased after 10 and 23 days. (**C**,**E**) glomerular HS expression was significantly reduced 10 and 23 days after induction of AN, as determined by immunofluorescence staining. (**D**) Representative pictures showing glomerular heparanase protein expression and (**E**) glomerular HS expression (magnification x400). 5–6 mice were used for analysis. *P<0.05, **P<0.01 and ***P<0.001 versus CTRL. CTRL, control; AU, arbitrary units.

To study the *in vivo* effects of eNOS deficiency on heparanase and HS expression, AN was induced in C57BL/6 WT, normally an AN resistant strain, and C57BL/6 eNOS-deficient mice. Fourteen days after the induction of AN, as expected, WT mice failed to develop proteinuria and had a normal renal function [4]. In contrast, eNOS-deficient mice developed overt proteinuria and had an impaired renal function [4]. Cortical heparanase mRNA expression (Fig 1A) and glomerular heparanase protein expression (Fig 1B and 1D) were normal in WT AN mice, whereas both heparanase mRNA expression and heparanase protein expression were significantly increased in the eNOS-deficient AN mice. In addition, glomerular HS expression was normal in the WT mice, but significantly reduced in the eNOS-deficient mice after induction of AN (Fig 1C and 1E). To evaluate whether AN caused similar effects on heparanase and HS expression in AN-sensitive mice as observed in the eNOS-deficient mice, AN was induced in the AN-sensitive Balb/c mice. We previously showed that eNOS expression was significantly reduced 24 hours after induction of AN in Balb/c mice [4]. By induction of AN, Balb/c mice develop proteinuria and renal damage after 10 and 23 days, as described before [21]. Cortical heparanase mRNA expression (Fig 2A) and glomerular heparanase protein expression (Fig 2B and 2D) were significantly increased 10 and 23 days after induction of AN, whereas glomerular HS expression was significantly reduced (Fig 2C and 2E). Together, these data indicate that eNOS prevents adriamycin-induced heparanase expression.

### The eNOS inhibitor ADMA increases transendothelial albumin passage in a heparanase-dependent manner

**Fig 3:**
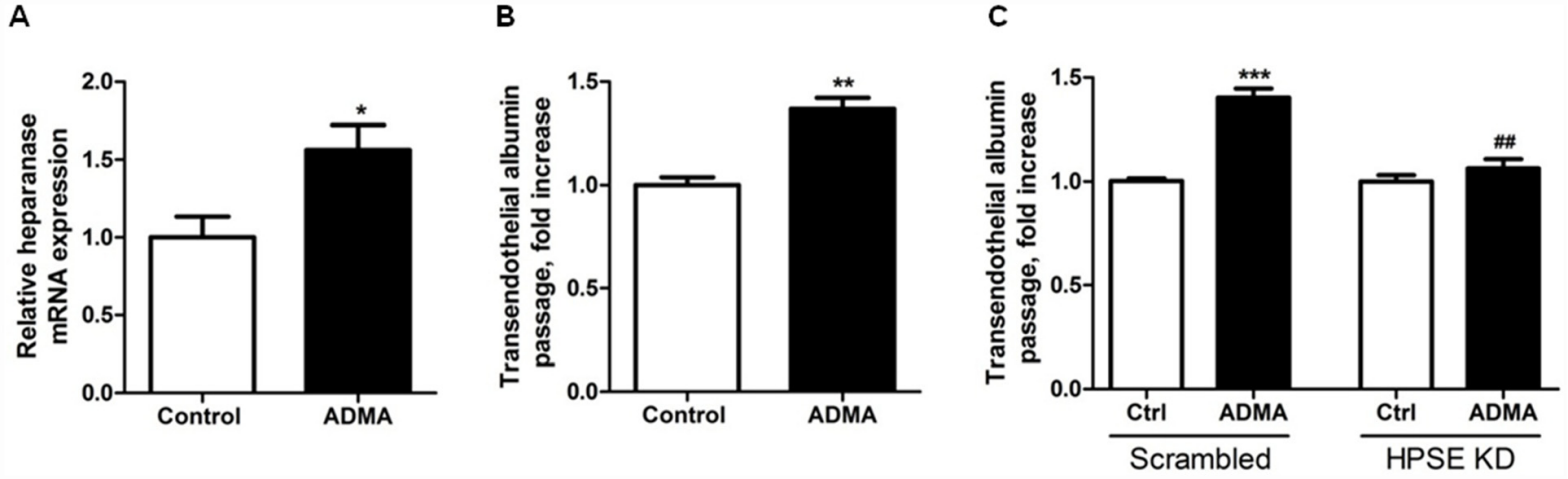
Inhibition of eNOS induces heparanase expression and increases transendothelial albumin passage in a heparanase-dependent manner in cultured mouse glomerular endothelial cells. (**A**) Treatment of mouse glomerular endothelial cells (mGEnC-1) with the eNOS inhibitor ADMA for 18 hours resulted in an increased heparanase mRNA expression. (**B**) Cumulative passage of FITC-labeled albumin across the mGEnC-1 monolayer (TEER: 28 Ω cm^2^) was increased 1.4-fold compared to control after treatment with ADMA for 18 hours. (**C**) Treatment of heparanase-silenced mGEnC-1 with ADMA for 18 hours led to lower transendothelial albumin passage compared with scrambled mGEnC-1 treated with ADMA. *P<0.05, **P<0.01 and ***P<0.001 versus control. ^##^*P*<0.01 versus ADMA scrambled. eNOS; endothelial nitric oxide synthase, ADMA; asymmetric dimethylarginine.

To extend the *in vivo* findings of eNOS deficiency on glomerular heparanase expression in AN, we evaluated whether eNOS regulates heparanase expression in glomerular endothelial cells *in vitro*. Treatment of mouse glomerular endothelial cells (mGEnC-1) with the eNOS inhibitor ADMA resulted in a 1.5-fold increased heparanase mRNA expression (Fig 3A). At the functional level, treatment of a monolayer of mGEnC-1 with AMDA increased transendothelial albumin passage 1.4-fold (Fig 3B). To show that the ADMA-induced increase in transendothelial albumin passage could be possibly mediated by heparanase, heparanase expression in mGEnC-1 was silenced with shRNA leading to ~60% reduced heparanase mRNA expression. Upon treatment with ADMA, transendothelial albumin passage was significantly lower in heparanase-silenced endothelial cells compared with endothelial cells transfected with a scrambled shRNA (Fig 3C). Taken together, these data indicate that eNOS inhibition increased heparanase expression and thereby transendothelial albumin passage.

## Discussion

Our data suggest that eNOS prevents the induction of glomerular heparanase expression and the development of proteinuria in AN. In addition, inhibition of eNOS activity in cultured mouse glomerular endothelial cells induces heparanase expression and increases transendothelial albumin passage in a heparanase-dependent manner.

Our study is the first to show that heparanase expression is regulated by eNOS. As outlined, eNOS and heparanase share regulating factors such as ROS, angiotensin II, PKC, AGEs, vitamin D and TNF-α [6,7,13,15–17,19]. However, not all of these aforementioned regulating factors are activators of eNOS and heparanase, nor solely operative in the endothelium, whereas none of these factors is specific for the glomerular endothelium. Interestingly, vitamin D is a positive regulator of eNOS, but a negative regulator of heparanase [7,19]. It has also been described that HS present in the endothelial glycocalyx is important for mechanosensing and eNOS-mediated NO production [25], which may suggest that a reduced glycocalyx thickness further hampers NO production, thereby further increasing heparanase expression. This self amplifying loop will ultimately lead to loss of endothelial glycocalyx and loss of NO production, and therefore endothelial dysfunction. We provided clear evidence for the interplay between eNOS and heparanase in AN, which may be operative in other glomerular diseases as well, since in the majority of glomerular diseases heparanase expression is increased, whereas glomerular HS expression is decreased [14]. Nevertheless, additional research is required to elucidate the complex regulation of both eNOS and heparanase, and their interplay, in glomerular diseases.

The regulation of heparanase expression by eNOS seems important for glomerular diseases, but may have important implications for other vascular beds outside the kidney as well. The vascular involvement in clinical manifestations associated with diabetes, sepsis, ischemia, artherosclerosis, and angiogenesis in cancer [26] may be dictated in part by the interplay between eNOS and heparanase. Future research should address the beneficial effects of a combined targeting of both eNOS and heparanase in experimental models of the aforementioned clinical manifestations and glomerular diseases.

Taken together, we postulate that a reduced glomerular expression/activity of eNOS increases heparanase expression, most likely due to a low NO level. This increased heparanase expression may explain a reduced thickness of the glomerular endothelial glycocalyx in (experimental) glomerular diseases, like diabetic nephropathy and adriamycin nephropathy [2,3].
